# How Could Medical and Surgical Treatment Affect the Quality of Life in Glaucoma Patients? A Cross-Sectional Study

**DOI:** 10.3390/jcm11247301

**Published:** 2022-12-08

**Authors:** Marco Rocco Pastore, Serena Milan, Rossella Agolini, Leonardo Egidi, Tiziano Agostini, Lorenzo Belfanti, Gabriella Cirigliano, Daniele Tognetto

**Affiliations:** 1 Eye Clinic, Department of Medicine, Surgery and Health Sciences, University of Trieste, 34129 Trieste, Italy; 2 Department of Economics, Business, Mathematics and Statistics, University of Trieste, 34100 Trieste, Italy; 3 Department of Life Sciences, University of Trieste, 34100 Trieste, Italy

**Keywords:** quality of life, canaloplasty, trabeculectomy, medical therapy

## Abstract

Background: To evaluate and compare the visual function and the quality of life (QoL) in glaucomatous patients treated with topical medical therapy (TMT) alone, canaloplasty (CP), or trabeculectomy (TB). Methods: A total of 291 eyes of 167 patients with primary open-angle glaucoma or secondary pseudoexfoliative glaucoma in TMT or surgically treated with CP or TB were included. Eligibility criteria for surgical patients included not needing TMT after surgery. Each patient underwent a visual field assessment and peripapillary retinal nerve fiber layer (pRNFL) optical coherence tomography and filled out the Glaucoma Symptoms Scale (GSS) questionnaire and the 25-Item National Eye Institute Visual Functioning Questionnaire (25-NEI-VFQ). Comparison between the QoL level of the three groups and its correlation with optic nerve’s anatomical and functional status was the primary outcome. Results: CP patients reported the best general vision (*p* = 0.01), a lower incidence of eye burning (*p* = 0.03), and the lowest annoyance level of non-visual symptoms (*p* = 0.006). QoL positively correlated with pRNFL thickness, whereas no correlation was found with visual field damage. Conclusion: CP provides a better QoL when compared both to TB and TMT, regardless of glaucoma stage. pRNFL seems to provide additional information for predicting change in QoL.

## 1. Introduction

Glaucoma is a neurodegenerative disease that causes a reduction of chromatic and contrast sensitivity, early alteration of light adaption, and the progressive development of characteristic visual field defects and optic disc damage [1,2,3,4]. This condition can lead to difficulties in performing daily activities such as reading, walking, or driving, limiting patients’ independence [5,6]. Moreover, the psychological impact can be fairly relevant: the loss of autonomy, together with the fear of going blind, may lead to depression, anxiety, and loneliness [7,8,9].

The main goal of glaucoma management is to preserve visual function (VF) and quality of life (QoL) [10]. QoL in glaucoma is assuming a leading role in healthcare, representing a significant index of glaucoma impact on patients and of health interventions’ effectiveness.

Glaucoma therapeutic options include medical and surgical treatment. Topical medical therapy (TMT) is generally the first approach in reducing intraocular pressure (IOP). However, it can lead to annoying local side effects such as irritation, burning, foreign body sensation, fatigue, blurred vision, dryness, photophobia, dry eye syndrome, allergies, and blepharitis [5,11]. Patients may also have difficulty applying eye drops and following complex treatment regimens. These issues can undermine patients’ satisfaction and their compliance with therapy [12,13]. Surgical therapy can reduce the incidence of these side effects; nevertheless, it is associated with specific unpleasant complications. Trabeculectomy (TB) represents the gold standard in glaucoma surgery, being the most effective surgical procedure for reducing IOP [13]. However, since it implies the creation of a communication between the anterior chamber and the subconjunctival space, it is burdened by numerous intraoperative and postoperative problems such as hypotony, bleb leakage, cataract development, choroidal hemorrhage, and infections [14,15,16]. Canaloplasty (CP) is a minimally invasive procedure requiring visco-dilatation of the Schlemm’s canal and the placement of an intracanalicular tension suture [17]. It has several advantages compared with TB, such as the absence of the filtering bleb and its complications, easier postoperative management, and faster recovery; however, a lower efficacy in reducing IOP was reported [15].

This study aimed to evaluate and compare the VF and the QoL in glaucoma patients treated with TMT, CP, and TB and to correlate it with anatomical and functional optic nerve alterations.

## 2. Materials and Methods

A cross-sectional study was conducted at the University Eye Clinic of Trieste between October and December 2020. The study protocol adhered to the tenets of the Declaration of Helsinki and was approved by the Institutional Review Board. The nature and the purpose of the investigation were fully explained, and informed consent was obtained from all participants.

Consecutive patients with a diagnosis of primary open angle glaucoma (POAG) or secondary pseudoexfoliative glaucoma (PEXG) in TMT or surgically treated with TB or CP by a single surgeon (DT) between January 2017 and July 2019 were included in the study. Patients were approached by the glaucoma specialist during regular clinic visits and screened for participation in this study. Glaucoma was diagnosed based on the presence of typical glaucomatous optic nerve head damage with focal or generalized neuroretinal rim thinning or cup/disc ratio asymmetry > 2 (in the absence of other neurodegenerative conditions) and associated with repeated corresponding glaucomatous visual field defects. Other eligibility criteria included age ranging between 55 and 80 years, previous cataract surgery, and for surgical patients to have at least 18 months (range, 19–35 months) of postoperative follow-up and no need for TMT after surgery.

Exclusion criteria were previous failed glaucoma surgery (cannulation failure during CP, secondary glaucoma surgery, or IOP > 18 mmHg [18] without topical glaucoma medication), previous eye intervention other than cataract surgery, and the presence of psycho-physical conditions interfering with the comprehension and the compilation of the questionnaires.

Enrolled patients underwent a complete ophthalmic examination and visual field assessment (standard automated perimetry, SAP) taken with the Humphrey Field Analyzer 3 (Carl Zeiss Meditec, Dublin, CA, USA) using the central 24-2 Swedish Interactive Threshold Algorithm strategy (SITA). Mean deviation (MD), pattern standard deviation (PSD), and Glaucoma Staging System 2 (GSS2) classification were registered [4].

The global average thickness (G) of the peripapillary retinal nerve fiber layer (pRNFL) was also registered via Heidelberg Spectralis II OCT (Software Version 6.15, Heidelberg Engineering, Heidelberg, Germany).

The number of different antiglaucoma topical medications applied daily by TMT patients was recorded.

At the end of the visit, the Italian versions of Glaucoma Symptoms Scale (GSS) [19,20,21,22] questionnaire and the 25-Item National Eye Institute Visual Functioning Questionnaire (25-NEI-VFQ) [23,24,25] were administered to each patient’s compilation according to the questionnaires’ specific compilation guidelines. Each eye was analyzed separately.

The GSS [19,20,21,22] questionnaire consists of ten questions related to ten eye complaints that are common in glaucoma patients; they are divided into two groups: six non-visual symptoms (burning, tearing, dryness, itching, irritation, feeling of foreign body) and four visual symptoms (blurred vision, difficulty seeing in daylight, difficulty seeing in darkness, halos around lights). Each symptom is analyzed in terms of presence and annoyance using a scale from 0 to 100 (0 indicates an intense symptom, and 100 corresponds to its absence): the higher the score, the greater the ocular wellbeing. Three scores are finally given: GEN is the total GSS score (mean of the ten subscale scores), SYM is the non-visual symptoms score (mean of the six subscales), and FUNC is the visual symptoms score (mean of the four subscales).

The 25-NEI-VFQ [23,24,25] consists of 25 main questions and an appendix of 13 additional items grouped in 12 subscales which investigate different fields of the vision-related QoL: general health, general vision, ocular pain, near activities, distance activities, social functioning, mental health, role difficulties, dependency, driving, color vision, and peripheral vision. Patients are required to estimate the fatigue encountered in performing a given daily activity, describing it as absent, small, moderate, severe, or so intense that they can’t carry it out. Answers are then converted into a numerical score, then the average for each subscale is calculated going from 0 to 100, where a higher score represents a better QoL.

### Statistical Analysis

Group homogeneity was checked via analysis of variance (ANOVA, *p* > 0.05) and proportion tests (*p* > 0.05). Quantitative variables were expressed in terms of mean ± standard deviations (SD). Regarding both questionnaires’ results, the comparison of CP and TB was assessed with the Wilcoxon test, whereas the Kruskal–Wallis test and G-test were required when analyzing CP, TB, and TMT. A *p*-value < 0.05 was considered statistically significant. Pearson’s correlation coefficient (*r*) was analyzed to study the correlation between QoL and the glaucoma stage (according to GSS2) and between QoL and G value; a corresponding correlation test was used to check the statistical significance.

Statistical analyses were performed using R software 3.6.1 (R Foundation for Statistical Computing, Vienna, Austria).

## 3. Results

A total of 291 eyes (145 right eyes, 146 left eyes) of 167 Caucasian patients (75 males and 92 females) met the inclusion criteria; the mean age of the study subjects was 77 years (SD, 8 years). Baseline characteristics of the three groups were similar in terms of age and gender according to ANOVA (*p* > 0.05) and proportion tests (*p* > 0.05). Out of 291 included eyes, 92 eyes (31.7%) underwent CP, 56 (19.2%) underwent TB, and 143 (49.1%) were treated with TMT. Regarding TMT patients, a mean number of 1.78 ± 0.74 different topical anti-hypertensive medications were instilled daily.

Visual field test results are reported in Table 1.

**Figure 1:**
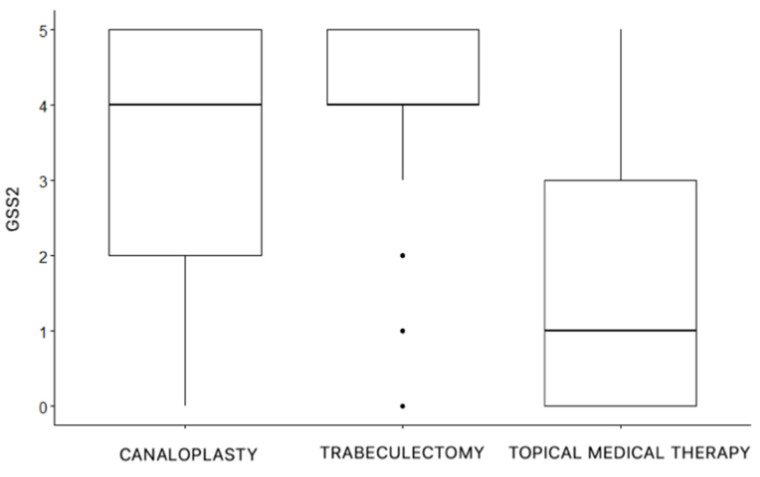
Visual field defect stages according to GSS2 in the three treatments groups.

The three groups were composed of patients affected by statistically significant different stages of glaucomatous visual field defect according to GSS2 (*p* < 0.001, ANOVA test), as shown in Figure 1. In addition, a statistically significant difference for MD, PSD, and GSS2 between the CP and TB groups was found (*p* < 0.001, ANOVA test).

As regards pRNFL, for the CP group, mean G was 60.51 μm ± 19 μm, for the TB group, mean G was 58.41 μm ± 20.2 μm, and for the TMT group, mean G was 75.71 μm ± 22.5 μm.

**Figure 2:**
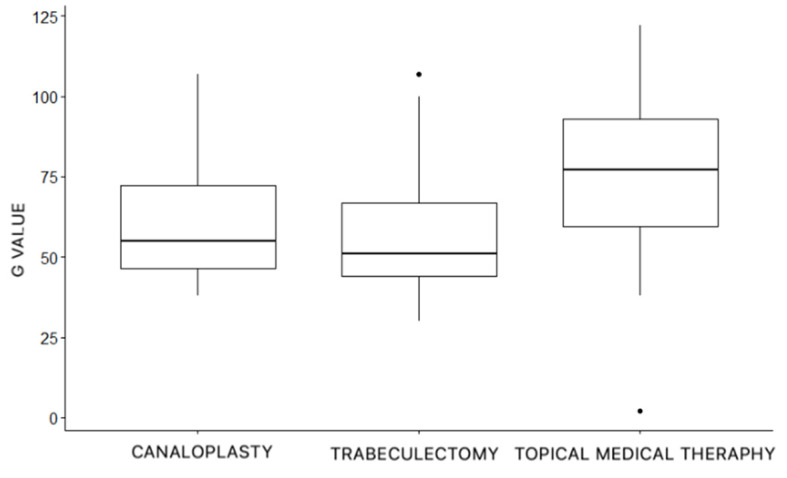
Global average thickness (G value) of the peripapillary retinal nerve fiber layer (pRNFL) in the three treatments groups.

The difference among the G values of the three groups was statistically significant (*p* < 0.001, ANOVA test), as shown in Figure 2.

**Figure 3:**
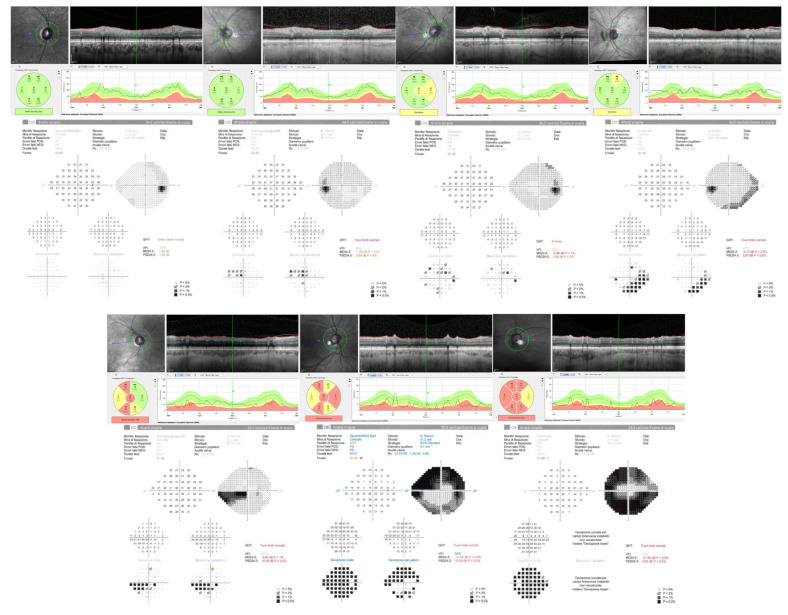
Peripapillary retinal nerve fiber layer and visual fields of patients with different glaucoma stages according to Glaucoma Staging System 2. From left to right, top: stage 0, borderline, 1, 2. From left to right, bottom: stage 3, 4, 5.

Visual field tests and pRNFL acquisitions images are shown in Figure 3.

**Figure 4:**
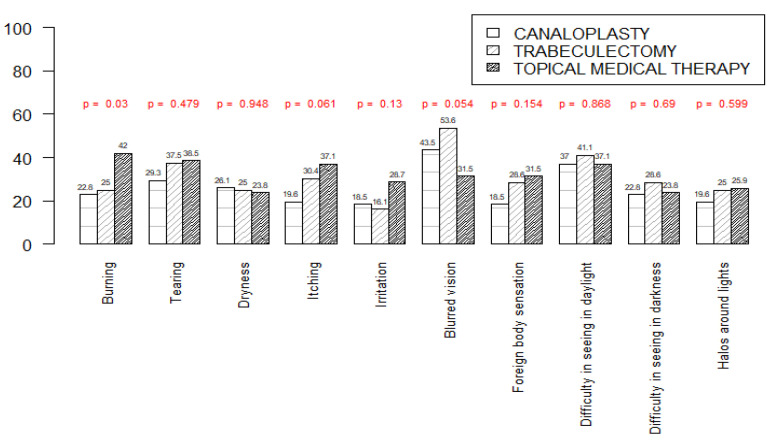
Incidence of the 10 symptoms analyzed by Glaucoma Symptom Scale (GSS) questionnaire in the three treatment groups. *p*-values resulting from the G-test are reported on the top of the columns.

GSS questionnaire results are reported in Figure 4, Table 2 and Table 3.

Firstly, the incidence of the 10 symptoms analyzed by the GSS questionnaire in the three treatment groups was analyzed (Figure 3); a statistically significant difference was noted in the incidence of “burning”, which was more frequently reported by TMT patients (*p* = 0.03, G test), whereas “blurred vision” was slightly correlated to TB treatment (*p* = 0.054, G test).

Annoyance of symptoms was also studied. Table 2 shows the comparison between CP and TB. CP patients referred a greater QoL: they reported higher (thus better) scores in SYM, FUNC and GEN; however, no statistically significant difference was found (Wilcoxon test).

Table 3 illustrates the comparison among CP, TB, and TMT group; CP patients reported a statistically significant lower annoyance of non-visual symptoms (*p* = 0.006).

The following parameters from the 25-NEI-VFQ were analyzed: general health, general vision, near vision, distance vision, mental health, and peripheral vision. Table 4 shows the comparison between CP and TB patients (Wilcoxon test).

CP patients reported higher and better scores in general vision than TB patients, and the difference was statistically significant (*p* = 0.005).

The comparison among CP, TB, and the TMT group (Kruskal–Wallis) is reported in Table 5.

The best results in general vision and peripheral vision were achieved by CP patients, whereas TB patients reported the lowest results; the differences were statistically significant (*p* = 0.01 and *p* = 0.04, respectively).

The Pearson correlation test was performed to assess the presence of a linear correlation between the QoL and functional and anatomical glaucomatous damage. Patients were considered as a single group, regardless of the type of treatment. No statistically significant correlation was found between QoL and the GSS2 stage. A weak positive correlation was found between pRNFL and the following subscales of the GSS questionnaire and the 25-NEI-VFQ: FUNC (*r* = 0.213, *p* < 0.001), general vision (*r* = 0.165, *p* = 0.0067), near activities (*r* = 0.244, *p* < 0.001), distance activities (*r* = 0.228, *p* < 0.001), mental health (*r* = 0.192, *p* = 0.0015), and peripheral vision (r = 0.27, *p* < 0.001).

Finally, a linear regression between general vision and MD, PSD, GSS2, and type of surgery was checked. We found a statistically significant regression coefficient (*p* = 0.0016) for type of surgery, with an improvement in the general vision of more than 10.3 points in CP compared to TB group.

## 4. Discussion

European Glaucoma Society’s guidelines state that the aim of glaucoma therapeutic management is the preservation of VF and of a good QoL [10]. According to the guidelines, medical therapy should be the first therapeutic approach. If it fails, surgical treatment is recommended. Currently, the gold standard of surgical therapy is represented by TB [10]. To avoid the above-mentioned complications, CP was developed; this surgical procedure is associated with a lower complication rate and it could represent both an effective and safe therapeutic strategy, which protects the surgeon from the burden of complications and preserves the patient’s wellbeing. This was confirmed by Klink et al. [26], who compared the 25-NEI-VFQ results of CP and TB patients, highlighting that VF was considerably better after CP; this kind of surgery was associated with a more preserved ability to read, watch television, see in the dark, and drive, having a lower impact on daily activities. Our study is consistent with this result; we found that CP guarantees a statistically significant better general vision score than TB (*p* = 0.005; Table 4). This is an encouraging outcome, since VF preservation constitutes the therapeutic goal to be achieved. CP does not require the presence of a filtering bleb, avoiding problems associated with it coming in touch with the cornea; this occurrence can cause keratopathy, tear film alterations, and eye surface damage, which can result in a worsening of visual performance [27,28].

In our study, we also evaluated the impact of TMT on QoL and compared it to the two above-mentioned surgical techniques. No available scientific study has provided a contextual comparison of these three therapeutic approaches yet; however, this evaluation could have important implications in clinical practice. Together with IOP reduction, surgery aims to preserve patients’ wellbeing and autonomy; it should lead to an improvement in patient QoL, since it virtually reduces the impact of ocular and systemic side effects of TMT, alleviating glaucoma interference in everyday life.

It has already been shown that TB surgery does not fulfill these purposes. In fact, Guedes et al. [29] compared the QoL of TMT patients with those who had undergone surgery (TB, laser iridotomy, and other techniques, but not CP), and those who required mixed therapy. The first group reported significantly better scores on the 25-NEI-VFQ, except for general health and driving, where the differences were not statistically significant. In the CIGTS (The Collaborative Initial Glaucoma Treatment Study) [30], 607 newly diagnosed patients were randomized to TMT or TB surgery and then underwent a 60-month follow-up, during which the impact on QoL of the two different therapies was assessed. One year after surgery, both ocular symptoms and VF were worse in the TB group.

In our study, according to GSS2 results, TMT was associated with the highest incidence of eye burning (*p* = 0.03), whereas blurred vision was more frequent in the TB group (*p* = 0.054). Moreover, CP patients reported the lowest annoyance of non-visual symptoms compared to TB and TMT patients, and the difference was statistically significant (*p* = 0.006).

As far as vision-related QoL is concerned, the 25-NEI-VFQ results show that CP is associated with greater values in the general vision parameter (*p* = 0.01) compared both to the TB group and the TMT group.

It was expected that CP surgery could be associated with a greater QoL than TB. Klink et al. [26] already showed that this minimally invasive and bleb-free procedure is linked to lower eye discomfort and a lower complication rate than TB; moreover, this technique does not require intraoperative and postoperative subconjunctival use of antiproliferative substances, whose application in TB surgery contributes to symptom development. As is known, the postoperative course could be challenging for each surgery; however, after CP, follow-up seems to be easier and most of the patients do not complain about eye discomfort, whereas TB is associated with longer hospitalization and follow-up, a higher chance of a second admission, and more frequent eye examinations (14.5% against 7.5% in canaloplasty) [26,27,28,31,32].

It was not expected that any surgery could guarantee a significantly better QoL than TMT; interestingly, in our study, CP seemed to achieve this goal. A likely reason for its supremacy can be found in the relationship between eye drops and Ocular Surface Disease (OSD). Pahljina et al. found that a reduction in the number of eye medications following glaucoma surgery (namely, phacoemulsification combined with the Xen gel stent) had a positive impact on patient QoL, according to the GSS questionnaire [33]. OSD affects 15% of healthy people over 65 years of age and 59% of glaucomatous patients; besides aging, one of the most important risk factors for its onset is eye drop instillation and years of treatment [11,34]. The damage is caused both by the active principle and the preservative. Benzalkonium chloride is the most widely used preservative; even at low concentrations, it exerts a toxic effect on the corneal-conjunctival surface, as it accumulates in the eye surface causing cell membrane lysis and altering corneal epithelial and Langerhans cell density; moreover, it determines a poorer tear production and interferes with the integrity of the superficial tear lipid layer, decreasing its stability, as demonstrated by the reduction in the break-up time [5,35].

Rossi et al. analyzed the relation between OSD and QoL in glaucoma patients receiving eye drops containing benzalkonium chloride through the 25-NEI-VFQ and the GSS questionnaires. Patients with OSD reached low mean total scores; the same results were obtained when dry eye syndrome was valued [11,35,36]. These conditions reduce visual performance and can limit daily activities such as reading, working on the computer, or driving. According to Van Gestel et al. [37], OSD seems to have a greater impact on QoL than the disease progression [36]; however, preservation of central and near vision, mobility, and daily activities is considered more important than the absence of eye discomfort, even by patients.

As regards peripheral vision, the TMT group gained the highest scores, and the difference turned out to be statistically significant (*p* = 0.04); this could be expected, since glaucoma visual field damage is typically centripetal, and this group was characterized by earlier GSS2 stages.

The supremacy of CP over TMT represents an interesting finding, considering that CP patients were affected by more advanced stages of anatomical and functional damage. Previous studies reported a correlation between the 25-NEI-VFQ results and visual field deterioration [7,20,38]. In our study, we found no correlation between the QoL and GSS2. Patients’ wellbeing seemed not to depend just on the progression of the perimetric defect; it represents a wider concept, influenced by numerous variables, which should be considered when evaluating the effectiveness of a given treatment. On the other hand, a linear positive correlation between QoL and pRNLF emerged. The relationship between anatomical changes and QoL is still not clear [39]. In a longitudinal study, Gracitelli et al. [40] already described how progressive thinning of RNFL was associated with a decrease in QoL over time. They reported that each 1-μm-per-year reduction in RNFL thickness corresponded to a modification in the 25-NEI-VFQ scores of 1.1 units per year; the association was confirmed even after accounting for visual field loss over time. This could partially explain why we found an anatomical correlation but not a functional one. Structural assessment seems to provide additional information for predicting change in QoL beyond what SAP can reveal; according to authors, there may be adjunctive visual changes that are relevant to QoL but cannot be fully captured by SAP, such as motion perception.

We also performed a correlation between the GSS questionnaire results and pRNLF; it is interesting to notice that a positive correlation was found for the visual symptoms (FUNC) subscale but not for the non-visual symptoms (SYM) subscale. Floriani et al. reported similar results when correlating GSS questionnaire’s results and GSS2 stages [7]. In our study, the impact of ocular disturbances (such as burning) on QoL shows no correlation with optic neuropathy, supporting our hypothesis that the type of treatment may have a role in determining patients’ wellbeing.

Limitations of the present study are its retrospective design and the small sample size. Moreover, as known, QoL can be affected by additional comorbidities, both ocular and systemic; nevertheless, since the three groups were homogeneous by sex and age, it is reasonable to assume that such pathologies also had a similar distribution. Future studies should aim to include a larger number of patients, have a prospective design, and a longer follow-up period.

## 5. Conclusions

CP seems to provide a better QoL when compared to both TB and TMT, guaranteeing better general vision, fewer symptoms, and a lower rate of complications. pRNFL thinning correlates with VR-QoL and seems to provide additional information on its changing besides what visual field test can reveal. However, local symptoms seem not to depend only on the structural damage, and the impact of treatment may be relevant. According to our findings, CP helps to combine IOP control with the need to ensure the patient’s wellbeing. Undergoing this kind of surgery, the patient will not have to instill topical drugs, avoiding their side effects; on the other hand, they will not face bleb-related ocular disorders, as in the case of TB. After a few weeks, the patient will be free from ocular discomfort. These findings suggest that CP could represent a valid therapeutic alternative in patients who poorly adhere to medical instruction or do not tolerate TMT; the lower rate of complications suggests it should be proposed more confidently even to younger patients, who will also benefit from the more delayed follow-ups.

## Figures and Tables

**Table 1:** Visual field test results in the three different treatment groups.

Parameters	Canaloplasty	Trabeculectomy	Topical Medical Therapy
MD ^1^	−13.65 ± 9.01	−16.88 ± 8.90	−6.70 ± 8.21
PSD ^2^	8.21 ± 4.35	8.33 ± 3.53	4.96 ± 3.92
GSS2 ^3^	3.36 ± 1.79	3.95 ± 1.45	1.84 ± 1.87

^1^ Mean Deviation. ^2^ Pattern Standard Deviation. ^3^ Glaucoma Staging System 2. Values are reported as mean ± standard deviation.

**Table 2:** Glaucoma Symptoms Scale (GSS) questionnaire: a comparison between canaloplasty and trabeculectomy through the Wilcoxon test.

Parameters	Canaloplasty	Trabeculectomy	*p*-Value (Wilcoxon)
GEN ^1^	83.3 ± 16.7	80.3 ± 16	0.12
SYM ^2^	87.6 ± 15.1	85.2 ± 13.4	0.09
FUNC ^3^	76.2 ± 27.9	73.8 ± 26.2	0.36

^1^ Total GSS score. ^2^ Non visual symptoms GSS score. ^3^ Visual function GSS score. Values are reported as mean ± standard deviation.

**Table 3:** Glaucoma Symptoms Scale (GSS) questionnaire: comparison between canaloplasty, trabeculectomy and topical medical therapy through the Kruskal–Wallis test.

Parameters	Canaloplasty	Trabeculectomy	Topical Medical Therapy	*p*-Value (Kruskal–Wallis)
GEN ^1^	83.3 ± 16.7	80.3 ± 16	78.4 ± 20.7	0.18
SYM ^2^	87.6 ± 15.1	85.2 ± 13.4	78.8 ± 22.7	0.006
FUNC ^3^	76.2 ± 27.9	73.8 ± 26.2	78.5 ± 24.5	0.41

^1^ Total GSS score. ^2^ Non visual symptoms GSS score. ^3^ Visual function GSS score. Values are reported as mean ± standard deviation. Statistically significant results are reported in bold text.

**Table 4:** General health, general vision, near vision, distance vision, mental health, and peripheral vision parameters of the 25-Item National Eye Institute Visual Functioning Questionnaire (25-NEI-VFQ): comparison between canaloplasty and trabeculectomy through the Wilcoxon test.

Parameters	Canaloplasty	Trabeculectomy	*p*-Value (Wilcoxon)
General health	68.3 ± 19.5	66 ± 17.1	0.38
General vision	73.2 ± 17.5	63 ± 21	**0.005**
Near vision	86.2 ± 21.9	86.1 ± 25.4	0.54
Distance vision	88.2 ± 18.1	89.3 ± 19.4	0.53
Mental health	78 ± 28.8	81.9 ± 23.3	0.73
Peripheral vision	88 ± 20.8	92 ± 21.2	0.20

Values are reported as mean ± standard deviation. Statistically significant results are reported in bold text.

**Table 5:** General health, general vision, near vision, distance vision, mental health and peripheral vision parameters of the 25-Item National Eye Institute Visual Functioning Questionnaire (25-NEI-VFQ): comparison between canaloplasty, trabeculectomy and topical medical therapy through the Kruskal–Wallis test.

Parameters	Canaloplasty	Trabeculectomy	Topical Medical Therapy	*p*-Value (Kruskal–Wallis)
General health	68.3 ± 19.5	66 ± 171	67.2 ± 20.1	0.67
General vision	73.2 ± 17.5	63 ± 21	68.1 ± 19.2	**0.01**
Near vision	86.2 ± 21.9	86.1 ± 25.4	90.1 ± 19.6	0.29
Distance vision	88.2 ± 18.1	89.3 ± 19.4	91.5 ± 15.2	0.46
Mental health	78 ± 28.8	81.9 ± 23.3	83.8 ± 23.9	0.37
Peripheral vision	88 ± 20.8	92 ± 21.2	93.7 ± 15.8	**0.04**

Values are reported as mean ± standard deviation. Statistically significant results are reported in bold text.

## Data Availability

Data are available on reasonable request by the corresponding author.
